# Evaluation of Lsa46 and Lsa77 Leptospiral Proteins for Their Immunoprotective Activities in Hamster Model of Leptospirosis

**DOI:** 10.1155/2018/1813745

**Published:** 2018-06-10

**Authors:** Aline F. Teixeira, Luis G. V. Fernandes, Antonio Souza Filho, Gisele O. Souza, Silvio A. Vasconcellos, Marcos B. Heinemann, Ana L. T. O. Nascimento

**Affiliations:** ^1^Laboratório Especial de Desenvolvimento de Vacinas, Instituto Butantan, Avenida Vital Brasil 1500, 05503-900 Sao Paulo, SP, Brazil; ^2^Laboratório de Zoonoses Bacterianas do VPS, Faculdade de Medicina Veterinária e Zootecnia, USP, Avenida Prof. Dr. Orlando Marques de Paiva 87, 05508-270 Sao Paulo, SP, Brazil

## Abstract

Leptospirosis is a neglected tropical disease caused by pathogenic* Leptospira* spp. The lack of an effective vaccine favors the increase of the disease. Currently, surface-exposed proteins are the main targets for the search of vaccine candidates. In this study, we examined whether the surface Lsa46 and Lsa77 proteins, previously identified as laminin and plasminogen binding proteins, have the capacity of inducing protection and sterilizing immunity against challenge with virulent* Leptospira* in hamster model. Animals were subcutaneously immunized with Lsa46, Lsa77, or a combination of both in Alum adjuvant and challenged intraperitoneally with* L. interrogans* serovar Kennewicki strain Pomona Fromm. Hamster immunization with Lsa46 or Lsa77 or both promoted a strong IgG response. Th2- and Th1-biased immune responses were observed when Lsa46 and Lsa77 were individually administered, respectively, as detected by the IgG1/IgG2/3 ratio. Immunized hamsters with the combined proteins induced a Th1-biased immune response. Although the immunization with Lsa46 and Lsa77 stimulated protective immunity with reduction of bacterial burden, when compared to animals individually immunized with the proteins, the data was not statistically significant. Thus, although promising, more studies are needed before the role of these proteins in stimulating sterilizing immunity in mammals is conclusively determined.

## 1. Introduction

Leptospirosis is a widespread zoonosis of human and veterinary concern. The global burden of leptospirosis is estimated to be over one million of annual cases [1]. In developing countries, outbreaks occur during rainfall periods, mainly in areas with inadequate infrastructure. This condition favors the proliferation of rats, the main reservoir of* Leptospira* [2]. Humans are generally infected through direct or indirect contact with the urine of infected animals [3]. Due to the undifferentiated symptoms, leptospirosis is commonly misdiagnosed for other acute febrile syndrome and most probably remains underestimated [3, 4].

Vaccines against leptospirosis are composed of whole inactivated bacteria cells, known as bacterins, or membrane preparations of pathogenic leptospires [5–7]. These vaccines induce short term-immunity, requiring annual boosts [8]. LPS are the basis of serological classification of* Leptospira* spp., being highly heterogeneous among leptospiral serovars. Accordingly, bacterin vaccines are protective only against the serovars included in the preparation [4]. Therefore, the development of conserved, cost-effective vaccines has been pursued.

Currently, subunit vaccines based on conserved outer membrane proteins (OMPs) have been the research focus of many groups worldwide [9–14], representing an important strategy for leptospirosis control. Several recombinant vaccines have been evaluated to date with variable efficacies [9–14], and in some cases, conflicting results have been observed [15, 16]. The most studied antigen candidates are LigA and LigB proteins, although animal protection and sterilizing immunity data in leptospirosis hamster model are controversial [15, 17–22]. Furthermore, LigA protein is not conserved among virulent leptospiral strains; its gene only presents in three* Leptospira* spp. [23]. Therefore, there is still a need for new vaccine targets.

In the present work, we evaluated the immune protective capacity of Lsa46 and Lsa77 recombinant proteins. Lsa46 and Lsa77 have OmpA-like domains and were previously characterized as laminin and plasminogen binding proteins. These proteins were localized at the cell surface by immunofluorescence assay and recognized by antibodies in leptospirosis serum samples suggesting their expression during infection [24]. The Lsa46 and Lsa77 proteins were individually or combined tested in leptospirosis animal model, and their ability in stimulating immune response is discussed.

## 2. Methods

### 2.1. Bacterial Strains

The virulent* L. interrogans* serovar Kennewicki strain Pomona Fromm (LPF) and* L. interrogans* serovar Copenhageni strain FIOCRUZ L1-130, culture-attenuated* L. interrogans* serovar Copenhageni strain M20,* L. borgpetersenii *serovar Whitcombi strain Whitcombi,* L. kirshneri *serovars Cynopteri strain 3522C and Grippotyphosa strain Moskva,* L. santarosai* serovar Shermani strain 1342K,* L. noguchii *serovar Panama strain 1342K, and saprophyte* L. biflexa* serovar Patoc strain Patoc1 were cultured at 28°C under aerobic conditions in liquid Ellinghausen-McCullough-Johnson-Harris (EMJH) medium (Difco, BD, Franklin Lakes, NJ, USA) containing 10% (vol/vol) rabbit serum. Virulent leptospire cultures are routinely maintained at the Faculdade de Medicina Veterinária e Zootecnia, Universidade de Sao Paulo (USP) in Sao Paulo, Brazil, by infection of Golden Syrian hamsters and subsequent bacterial isolation from kidney.* Escherichia coli* BL21 DE3 Star pLysS cells were used as recombinant protein expression hosts.

### 2.2. Recombinant Proteins Purification

Lsa46 and Lsa77 expression and purification steps are detailed in [24]. Briefly,* E. coli* BL21 DE3 Star pLysS cells containing each recombinant plasmid were grown in LB medium (Luria–Bertani) containing 50 *μ*g/mL ampicillin to an OD600 nm of approximately 0.6 and the expression of the recombinant proteins was induced by 1 mM isopropyl *β*-d-1-thiogalactopyranoside (IPTG) for 3 h under constant agitation at 37°C. The cells were harvested by centrifugation and the resulting bacterial pellet, after sonication, was resuspended in a buffer containing 20 mM Tris-HCl (pH 8.0), 500 mM NaCl, and urea 8 M. The proteins, expressed with a N-terminus 6xHis, were purified through a Ni2+ charged chelating fast-flow chromatographic column (GE Healthcare, Buckinghamshire, UK) and refolded as previously described [24].

### 2.3. Leptospiral DNA Extraction and PCR Analysis

Approximately, 3x10^8^ leptospiral cells were harvested by centrifugation at 8,000 x g for 15 min. Pellet was resuspended in 467 *μ*L TE buffer (10 mM Tris-HCl, pH 7.4; 1mM EDTA, pH 8.0) 30 *μ*L SDS (10%) and 3 *μ*L proteinase K (20 mg/mL) (Sigma) and incubated for 1h at 37°C. After, 500 *μ*L phenol:chloroform solution (1:1) was added and the solution was thoroughly mixed. Samples were centrifuged at 12,000 x g for 5 min and the aqueous phase was transferred to a new tube. The phenol: chloroform extraction was repeated. It was added 1:10 volume of 3M sodium acetate and 0.6 volume of isopropanol the aqueous phase and the solution was incubated for 1h at -20°C. After, samples were centrifuged at 12,000 x g for 20 min, the pellet was washed with 1mL of 70% ethanol and recovered by centrifugation at 10,000 x g for 10 min at 4°C. The air-dried pellet was then resuspended in 50 *μ*L of water. The DNA fragments were amplified using oligonucleotides designed according to* L. interrogans *serovar Copenhageni genome sequences. PCR was performed in a reaction volume of 50 *μ*L containing 1x PCR buffer, 2mM MgCl_2_, 20 pmol of each specific primer, 200 *μ*M of dNTPs, 100 ng of genomic DNA, and 1 U Taq DNA Polymerase. Cycling conditions were 94°C for 4 min, followed by 35 cycles at 94°C for 50 sec, 60°C for 50 sec, 72°C for 2 min, and a final extension cycle of 7 min at 72°C. PCR amplified products were analyzed on a 1% agarose gel.

### 2.4. Leptospiral RNA Extraction and cDNA Generation

Leptospiral cells at late-log phase contained in 30 mL EMJH medium were collected by centrifugation (8,000 x g for 15 min) in RNase-free tubes and resulting pellet was resuspended in 1 mL Trizol reagent (Invitrogen), vortexed, and incubated for 10 min at room temperature. After, 260 *μ*L chloroform was added; mixture was vigorously shaken and incubated at room temperature for 10 min. Samples were centrifuged at 12,000 x g for 15 min at 4°C for phase separation; the aqueous phase was transferred to a new tube and 660 *μ*L of isopropanol was added for nucleic acid precipitation, performed at room temperature for 10 min. Then, tubes were centrifuged (12,000 x g for 10 min, 4°C) and the resulting pellet was washed with 1.35 mL of 75% ethanol, diluted in RNase-free water, and recovered by centrifugation at 7,500 x g for 5 min at 4°C. The supernatant was removed and the pellet air dried for approximately 30 min and then resuspended in 44 *μ*L of RNase-free water and incubation at 55°C for 10 min. Residual DNA was eliminated by incubation with DNase I (Invitrogen). Samples quality was accessed by BioSpectrometer® basic (Eppendorf) quantification and visualization at 1% agarose gel. For obtaining leptospiral cDNA, the iScript cDNA synthesis Kit (Biorad) was employed. Briefly, two *μ*L of total extract RNA was mixed with 4 *μ*L of 5X iScript reaction mix and 1 *μ*L iScript RT to a final volume of 20 *μ*L. Reactions were incubated at 25°C for 5 min, followed by 45 min incubation at 42°C and then 5 min at 85°C for inactivating the enzyme. A control lacking the reverse transcriptase (RT-) was also employed to rule out genomic DNA contamination in the samples.

### 2.5. Detection of Gene Transcripts by q-PCR

Messenger RNAs (mRNAs) were indirectly verified by real-time PCR cDNA amplification with specific primers to LIC13479 and LIC10050 (Lsa46 and Lsa77 respectively) coding sequence. RT-qPCRs were performed using the Applied Biosystems 7300 Real-Time PCR system equipment and SYBR green to detect the synthesized dsDNAs. Standard curves were performed in order to obtain the concentration and amplification efficiency of the primers to be used. RT-qPCR was performed in a reaction volume of 25 *μ*l containing 25 ng of cDNA template, 300 nM each oligonucleotide (‘F' and ‘R'), and 12.5 *μ*l SYBR Green PCR Master Mix (Applied Biosystems) as recommended by the manufacturer. All reactions were performed in triplicate in 96-well optical plates. Negative controls using all the reagents except the cDNA were run in parallel (NTC, no template control), as RT- samples. Cycling conditions were 50°C for 2 min, 95°C for 10 min, followed by 40 cycles of 95°C for 15 seconds, and 60°C for 30 s. Dissociation curves were obtained after each reaction by heating the PCR products from 60 to 95°C, in order to verify the specificity of reaction. The relative gene expression among leptospiral was analyzed using the comparative 2^−ΔΔCT^ method and the 16S gene was used as the internal control to normalize the gene expression.

### 2.6. Animal Immunization and Lethal Challenge Assays

Male hamsters (6–8 weeks old) were immunized subcutaneously with 300 *μ*l emulsion volumes containing 50 *μ*g of each recombinant protein alone or with 25 *μ*g of each combined, mixed with 12.5% Alhydrogel (2% Al(OH)3) as adjuvant. One booster injection was given after 2 weeks with the same preparation of recombinant protein. In the negative control group, hamsters were injected with PBS in 12.5% Alhydrogel. Animals immunized with heat-killed whole-leptospires (bacterin vaccine) were included as positive control of survival. Hamsters were immunized with a dose composed of 10^9^ inactivated leptospires in 12.5% Alhydrogel on day 0 and 14. After immunization, animals were challenged intraperitoneally with an inoculum containing a total of 10^4^ leptospires, previously defined by a survival curve experiment [25]. Hamsters received water and food* ad libitum* and were monitored daily for clinical signs of leptospirosis and euthanized when clinical signs of terminal disease appeared. Hamsters surviving on day 21 after challenge were euthanized. Hamsters in each group were bled from the retro-orbital plexus after each immunization and the sera stored. Two independent experiments were performed, involving groups of 6-10 animals each. Animals that survived lethal challenge were sacrificed and their kidneys removed aseptically and then macerated and inoculated in EMJH medium for bacterial growth monitoring.

### 2.7. Evaluation of the Humoral Immune Response

Sera of immunized animals were analyzed by ELISA for antibody titers determination: total IgG and its subclasses IgG1 and IgG2/IgG3. ELISA plates were coated with 250 ng of each recombinant protein, and wells were blocked with PBS-T containing 10% nonfat dry milk and incubated with different dilutions of hamster sera, ranging from 1:200 to 1:409,600. Plates were washed and incubated either with HRP-conjugated anti-hamster total IgG (1:5,000, Sigma), anti-hamster IgG1, or anti-hamster IgG2/IgG3 (1:5,000, Southern Biotechnology). The wells were washed three times, and o-phenylenediamine (1 mg/ml) in citrate phosphate buffer (pH 5.0) plus 1 *μ*l/ml H_2_O_2_ was added (100 *μ*l per well). The reaction was allowed to proceed for 10 min and was interrupted by the addition of 50 *μ*l of 2 M H_2_SO_4_ to the mixture. Readings were taken at 492 nm with a microplate reader (Multiskan EX; Thermo Fisher Scientific, Helsinki, Finland). The titer was considered the maximal dilution that showed an OD492 nm value above 0.1.

### 2.8. Statistical Analysis

The statistical analysis was performed using Graph Prism 5 (GraphPad Software). The Log-rank (Mantel-Cox) test and Fisher's exact test were used to compare survival curves among experimental groups. The statistical significance was considered to p-value <0.05.

### 2.9. Ethics Statement

All animal studies were approved by the Ethical Committee for Animal Research of Instituto Butantan and of School of Veterinary Medicine and Animal Science, University of Sao Paulo, Sao Paulo, SP, Brazil, under protocols 890/12 and 3158/13, respectively. The Committees in Animal Research adopt the guidelines of the Brazilian College of Animal Experimentation.

## 3. Results

### 3.1. Distribution of Lsa46 and Lsa77 among* Leptospira* Strains

**Figure 1:**
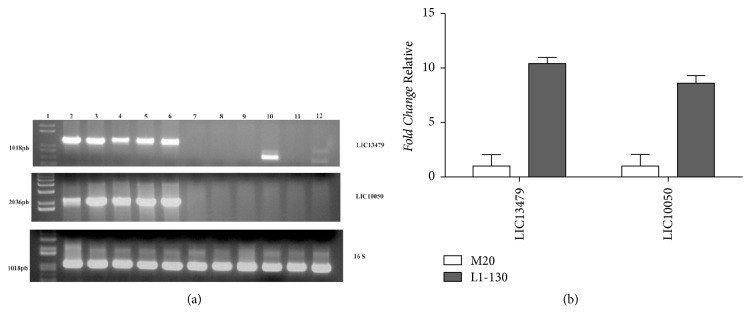
**Distribution and expression of LIC13479 and LIC10050 genes in* Leptospira *spp.** (a) Genomic DNA from* L. interrogans *serovars: Copenhageni (1), Canicola (2), Icterohaemorrhagiae (3), Pomona (4), Hardjo (5),* L. borgpetersenii *serovar Whitticombi (6),* L. kirshneri *serovars Cynopteri (7) and Grippotyphosa (8),* L. santarosai *serovar Shermani (9),* L. noguchii *serovar Panama (10), and saprophytic species* L. biflexa *serovar Patoc (11) were subjected to PCR analysis with specific primers. The integrity of the DNA used was determined by amplification of 16S DNA. (b) Transcript levels of the LIC13479 and LIC10050 were determined by qRT-PCR in virulent* L. interrogans *serovar Copenhageni strain FIOCRUZ L1-130 and culture-attenuated* L. interrogans *strain M20. The relative gene expression was calculated by 2^−ΔΔCT^ method after normalization with* 16S. *Error bars show the mean ± SD from three independent experiments performed in triplicate.

PCR analysis of the genes LIC13479 (Lsa46) and LIC10050 (Lsa77) identified their presence in five different* L. interrogans *serovars: Copenhageni, Canicola, Icterohaemorrhagiae, Pomona, and Hardjo (Figure 1(a)). DNA fragments of both genes were not amplified in the nonpathogenic strain* L. biflexa*, and in the pathogenic species* L. borgpetersenii* serovar Whitticombi,* L. kirsheneri* serovars Cynopteri and Grippotyphosa, and* L. santarosai* serovar Shermani. Only the gene LIC13479 was detected in* L. noguchii *serovar Panama. Amplification of the 16S DNA was employed as DNA integrity control (Figure 1(a)). BLAST analysis confirmed that both coding sequences exhibit 99 or 100% of identity with the five serovars of* L. interrogans* experimentally tested (Table 1). However,* in silico* amino acid sequence analysis showed that LIC13479 and LIC10050 are present with high percentage identity in other pathogenic species:* L. kirschneri *serovars Cynopteri and Grippotyphosa,* L. santarosai* serovar Shermani, and* L. noguchii* serovar Panama. The data suggest that these coding sequences exhibit a broader spectrum of conservation among leptospiral pathogenic strains than the ones depicted in Figure 1(a). Only 44-45% conservation was detected in the saprophyte* L. biflexa* strain (Table 1). The discrepancy of our data might be due to different bacterial isolates. Nevertheless, the data point out that these proteins represent potential targets worth to be tested in leptospiral challenge assays.

### 3.2. Expression of Lsa46 and Lsa77 in* L. Interrogans* Serovar Copenhageni

To evaluate the expression of LIC13479 (Lsa46) and LIC10050 (Lsa77) genes in virulent* L. interrogans* serovar Copenhageni strain FIOCRUZ L1-130 and culture-attenuated* L. interrogans* strain M20, RT-qPCR experiments were performed. The results show the presence of LIC13479 (Lsa46) and LIC10050 (Lsa77) transcripts in both strains tested. The relative higher expression detected with both transcripts in the virulent L1-130 compared to the culture-attenuated M20 strain indicates that Lsa46 and Lsa77 may have a role in leptospiral virulence (Figure 1(b)).

### 3.3. Assessment of Humoral Immune Response Induced in Hamsters Inoculated with Lsa46 Protein

**Figure 2:**
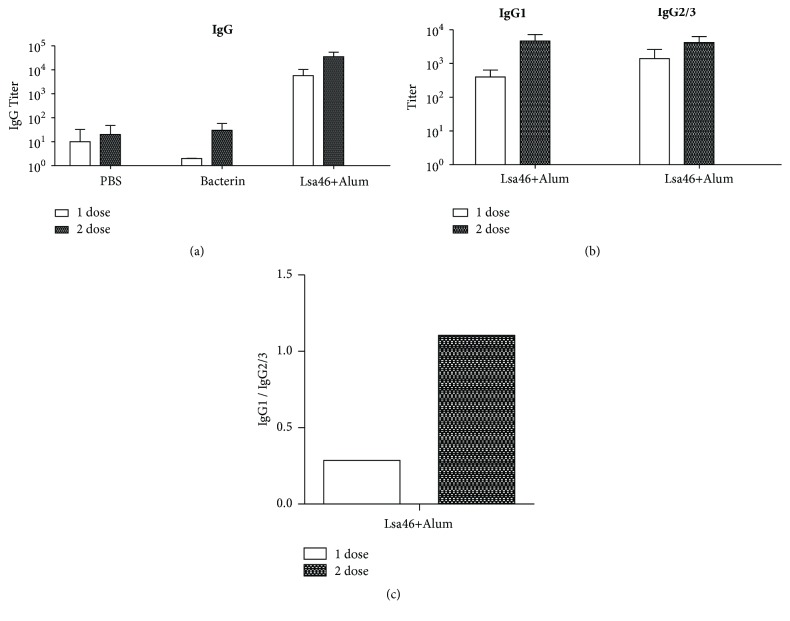
**Immune response induced by immunization with Lsa46.** Hamsters were inoculated subcutaneously with Lsa46 combined with 12.5% Alum and booster was administered after two weeks. Immunized animals with PBS or bacterin were used as negative and positive control, respectively. Animals were bled two weeks after each immunization and sera were used to determine antigen-specific total IgG by ELISA (a) and IgG1 and IgG2/3 (b). The IgG1/IgG2/3 ratios are shown in (c). Error bars show the mean ± SD from two independent experiments performed in triplicate. Representative data are shown.

To assess the antibody immune response elicited in hamsters by Lsa46 protein, sera from animals subcutaneously immunized with Lsa46 were used in an indirect ELISA for total IgG measurements. Animal serum samples were collected 2 weeks after each immunization (15 and 30 days). The results refer to two independent experiments (Figure 2). In both assays, immunization with Lsa46 recombinant protein in Alum promoted higher titers of total IgG compared to the ones induced by bacterin (Figure 2(a)). To evaluate the polarity of the immune response against Lsa46, antigen-specific IgG1 and IgG2/3 subtypes responses were also measured. The results show that the levels of both IgG1 and IgG2/3 increased in the sera of animals immunized with the Lsa46 (Figure 2(b)). The IgG1:IgG2/3 ratio points to a mixed immune response, with a higher trend for Th2 immune response pattern (Figure 2(c)).

### 3.4. Humoral Immune Response Induced by Lsa77 Protein Administration in Hamsters

**Figure 3:**
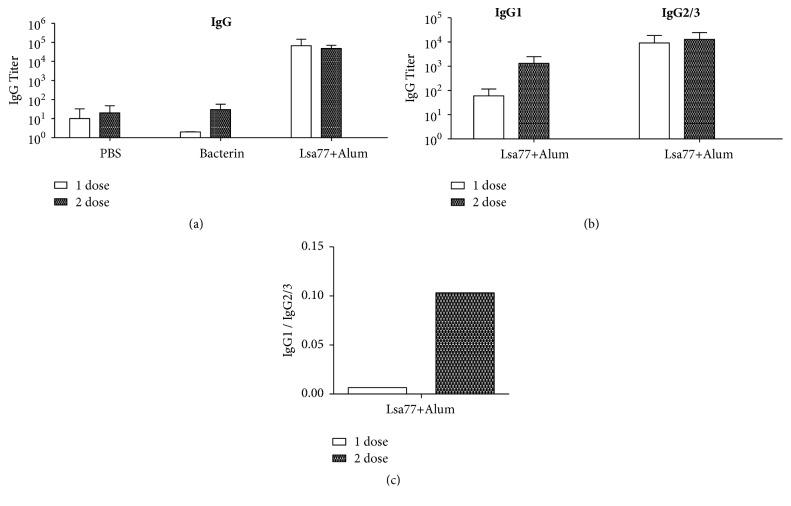
**Antibodies induced by immunization with Lsa77.** Hamsters were immunized subcutaneously with Lsa77 combined with 12.5% Alum and booster was administered after two weeks. Inoculated animals with PBS or bacterin were used as negative and positive control, respectively. Animals were bled two weeks after each immunization and sera were utilized to determine antigen-specific total IgG (a) and IgG1 and IgG2/3 (b) response by ELISA. The IgG1/IgG2/3 ratios are shown in (c). Error bars show the mean ± SD from two independent experiments performed in triplicate. Representative data are shown.

Likewise, indirect ELISA for total IgG measurements was also performed to assess the antibody immune response promoted in hamsters inoculated with Lsa77. Animal serum samples were collected 2 weeks after each immunization (15 and 30 days), and the results present are from two independent experiments (Figure 3). In both experiments, Lsa77 recombinant protein plus Alum induced higher titers of total IgG compared to the ones elicited by bacterin (Figure 3(a)). Similarly, we examined the polarity of the immune response against Lsa77, measuring antigen-specific IgG1 and IgG2/3 subtypes. Contrary to the results observed with Lsa46, immune response induced by Lsa77 shows titer levels of IgG2/3 higher than IgG1 in both experiments (Figure 3(b)). The IgG1:IgG2/3 ratio points to a Th1 immune response (Figure 3(c)).

### 3.5. Evaluation of Antibody Response in Hamsters Immunized with a Combination of Lsa77 and Lsa46 Proteins

**Figure 4:**
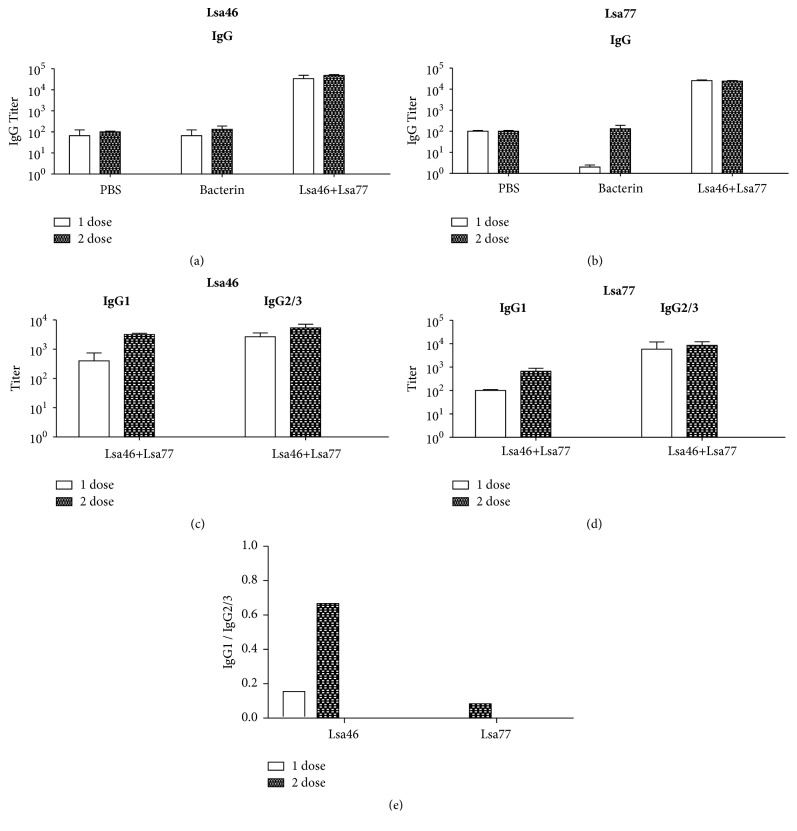
**Humoral immune response induced by immunization with the combined recombinant proteins.** Hamsters were immunized subcutaneously with Lsa46 and Lsa77 combined with 12.5% Alum; booster was administered after two weeks. PBS or bacterin immunized animals were used as negative and positive control, respectively. Animals were bled two weeks after each immunization and sera were utilized to determine Lsa46-specific total IgG, IgG1 and IgG2/3 response ((a) and (c)) or Lsa77-specific response ((b) and (d)). The IgG1/IgG2/3 ratios are shown in (e). Error bars show the mean ± SD from two independent experiments performed in triplicate. Representative data are shown.

The diverse immune response trend elicited in hamsters individually inoculated with Lsa46 or Lsa77 prompted us to investigate what type of antibody response would result by administration of both proteins. Similarly as evaluated for each protein, animal serum samples were collected 2 weeks after each immunization (15 and 30 days), and an indirect ELISA was used for total IgG measurements. We employed Lsa46 as a coating protein, and Figures 4(a), 4(c), and 4(e) illustrate the data of a representative experiment of two independent performed experiments. Likewise, Lsa77 was used as a coating protein and Figures 4(b), 4(d), and 4(e) show the results of a representative experiment of two independent performed experiments. Animals immunized with combined Lsa46+Lsa77 in Alum also exhibited high levels of total IgG, either using Lsa46 (Figure 4(a)) or Lsa77 (Figure 4(b)), as a probe. The polarity of the immune response against both proteins, measuring antigen-specific IgG1 and IgG2/3 subtypes, shows higher titer levels of IgG2/3 than IgG1 in both experiments, either probing with Lsa46 (Figure 4(c)) or Lsa77 (Figure 4(d)). In both cases, the IgG1:IgG2/3 ratio points to a Th1 polarized immune response (Figure 4(e)). Interestingly, the higher levels of IgG2/3 observed in serum samples of animals immunized with both proteins suggest that the presence of Lsa77 was capable of promoting an immune modulation towards Th1, since immunization with Lsa46 protein alone promoted higher titers of IgG1 (Figure 2(b)).

### 3.6. Immune Protection of Hamsters Induced by Lsa46 and Lsa77 Recombinant Proteins in Leptospirosis Challenge Assays

**Figure 5:**
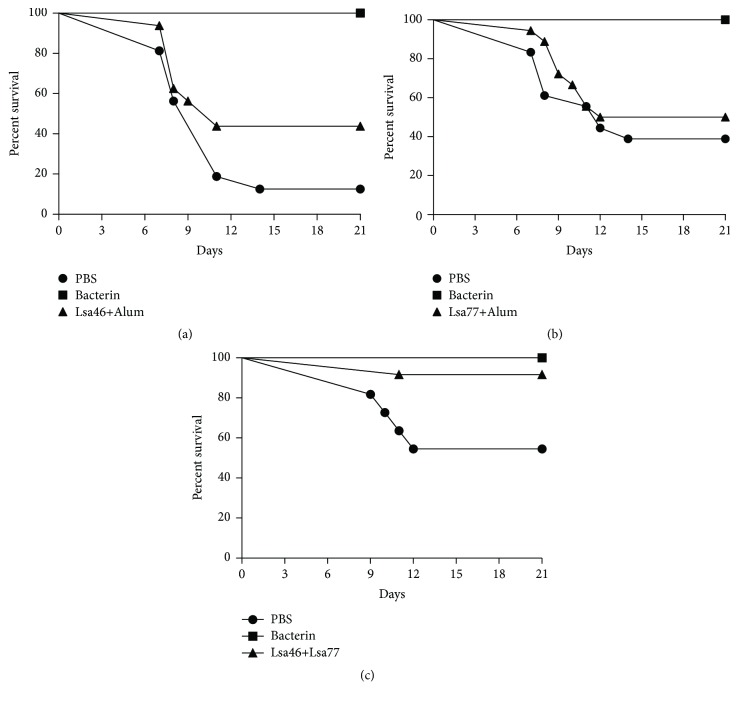
**Protective effect induced by immunization with Lsa46 and Lsa77.** Animals were immunized subcutaneously with Lsa46 (a), Lsa77 (b), or combined Lsa46 and Lsa77 (c) with 12.5% Alum; booster was administered after two weeks. Inoculated animals with PBS or bacterin were used as negative and positive control, respectively. Two weeks after the second immunization, animals were challenged intraperitoneally with 1x10^4^* L. interrogans *serovar Kennewicki strain Pomona Fromm. After challenge, hamsters were monitored for 21 days. Two independent experiments were performed and pictures represent the data of experiments 1 and 2. Rates of survival were compared using log-rank and Fisher's exact test. P-value < 0,05 was considered significant.

The protective efficacy of the Lsa46 and Lsa77 recombinant proteins was evaluated in lethal challenge in two independent experiments. At 28 days after i.p. inoculation, survival in animals immunized with Lsa46/Alum exhibited 66% and 30% of protection in the first and second experiment, respectively. The positive control group afforded 100% protection in both experiments, while 0% and 20% survivors were observed in the PBS-control group, in experiments 1 and 2, respectively. The statistical analyses are presented in Table 2, significance protection is observed only in the first experiment. When the percentage of protection was calculated using the total number of survivors of the two experiments, 44% of protection was afforded by Lsa46, versus 13% of survivors in PBS-injected control animals (Figure 5(a) and Table 2). Furthermore, renal colonization was observed in the animals immunized with Lsa46 (Table 2), indicating that immune protection was not sterilizing. Immunization with Lsa77 recombinant protein conferred 50% survival in both experiments. However, no statistically significant difference was found when compared to PBS-control group (Table 3). When the percentage of protection was calculated using the total number of survivors of the two experiments, 50% of protection was afforded by Lsa77, against 39% of survivors in PBS-injected control animals (Figure 5(b)). Animals immunized with Lsa77 did not exhibit reduction in renal colonization when compared to PBS-control group (Table 3).

To assess the protective capacity of the combined Lsa46 and Lsa77 recombinant proteins, animals were immunized with 25g of each protein mixed with Alum. The results obtained of two experiments reveals that Lsa46+Lsa77 group afforded 100 and 90% survivors, in experiments 1 and 2, respectively (Table 4), while 66 and 50% of animals survived in the PBS-control group in the same experiments (Table 4). Thus, the difference of about 40% in the survival of animals immunized with Lsa46+Lsa77 when compared to PBS-injected group was not statistically significant (Figure 5(c)). However, among the vaccinated survivors the number of animals positive for the presence of leptospires in the kidneys was lower than the control group (Table 4), indicating the potential of this formulation to eliminate leptospiral colonization. Thus, the stimulated immune response by Lsa46+Lsa77 combined formulation improved the animal protection, but it was not enough to achieve a complete sterile protection.

## 4. Discussion

Presently, the available vaccines against leptospirosis are based on inactivated whole-cell or membrane preparation. Although these vaccines provide full protection, they fail to induce immune response memory and consequently require annual vaccination. Further, these vaccines show high side-effects and elicit a minor or no cross protection against serovars not included in the preparation [4]. Therefore, the development of cost-effective and safe vaccine, capable of providing cross protection and long-lasting immunity is needed. Leptospiral genome sequences availability allowed the* in silico* analysis of sequences with potential for vaccine development.

We have characterized Lsa46 and Lsa77 recombinant proteins and show that they are laminin and plasminogen binding proteins, located at the leptospiral surface. Bioinformatics analysis of the Lsa46 and Lsa77 coding sequences showed that they are conserved in several pathogenic strains of* Leptospira *[24]. In this work, we have completed the description of these proteins and examined their potential to induce humoral and protective immune response in hamsters.

PCR analysis confirms the genomic conservation among* L. interrogans *serovars. Furthermore, analysis by qPCR indicates that the genes are more expressed in virulent* L. interrogans* L1-130 than culture-attenuated M20 strains. Generally, conserved proteins containing multiple B and T-cell epitopes are pivotal for the development of broad-spectrum vaccine. Indeed, Lsa46 and Lsa77 proteins showed a strong immunogenicity in hamsters, resulting in the production of high levels of antibodies. Hamster serum raised against each recombinant protein was able to recognize the homologous protein and demonstrate cross-reaction against the others, possibly due to the presence of OmpA-like domain (data not shown). Serum cross-reactivity has been observed with the LIC10507, LIC10508, and LIC10509 outer membrane proteins from* Leptospira *[26]. Despite these interesting features [26–28] their immune protective capacity has never been investigated.

The most studied and promising candidates to date are the leptospiral immunoglobulin-like (Lig) proteins. It has been reported that Lig proteins are localized to the bacterial surface and considered major antigens recognized during the acute host infection [29]. The immune protective activity of LigA and LigB of* L. interrogans* serovar Manila against a challenge with a homologous serovar in C3H/HeJ mice was reported [17]. The protective capacity of LigA has also been shown either by recombinant protein or as DNA vaccine against challenge in hamsters [18, 30, 31]. Oral immunization with* E. coli* expressing lipidated LigA showed protection in hamsters challenge [21]. However, in all cases mentioned, protection afforded by LigA was not sterilizing. However, Lucas and colleagues [32] have demonstrated that although LigA protein eliciting antibody responses, hamsters were not protected against infection. Due to its low conservation among pathogenic* Leptospira* species, LigA is not considered a good antigen candidate for the development of a broad-spectrum vaccine [33]. Immunogenicity and protective efficacy of recombinant LigB in a hamster challenge model have been shown [20, 34]. Immunization with a DNA vaccine encoding LigB (amino acids 131 to 645) elicited 62.5% (5/8) protection of hamsters and conferred sterilizing immunity in 80% of the surviving hamsters [35]. Lately, it has been shown that rLigB (131–645)/in Alum has the capacity to promote full protection against lethal challenge in the hamster model of acute leptospirosis. Moreover, this antigen conferred sterile immunity in survival animals [16].

Immunoprotective capacity of recombinant LigA and LigB proteins has been recently revisited, and conflicting data were achieved [15]. They showed that hamsters immunized with recombinant LigA 7'-13 afforded animal protection from death but not against infection, while administration with LigB 0-7 conferred only partial animal protection and was positive for renal colonization. Hamsters immunized with a combination of both afforded 100% protection but did not confer sterilizing immunity. Thus, it seems that although promising, more investigation is needed before vaccine development with Lig proteins could be established.

The protective role of Lsa46 and Lsa77 proteins when individually evaluated in hamster model showed that each protein afforded partial protection and failed to elicit bacterial clearance in the kidneys. Similar results were obtained when other recombinant proteins were administered in hamsters, including the Lsa66 OmpA-like domain protein [11, 36]. OmpA-like domain is similar to OmpA outer membrane protein from* E. coli* and other Gram-negative bacteria. OmpA is conserved across different species and seems to be involved in many functions, which suggests it has an important functional role in bacteria [37–39]. Furthermore, the OmpA protein of several pathogens has been considered as potential vaccine candidates [40–42]. In* Leptospira*, in addition to Lsa66 protein, another three OmpA-like proteins were assayed as vaccine candidates for leptospirosis. However, these proteins failed to promote sterilizing immunity [11, 43].

The protective effect of immunization with Lsa46 and Lsa77 seems to be synergistic, since an increased level of protection with reduced kidney bacterial burden was observed when compared to animals immunized with either Lsa46 or Lsa77 alone. The data, however, were not statistically significant, due to the high survival rate detected in control animals. Similar results are reported in the literature, with survival rate in control animals ranging from 13 to 75% [18, 44–47]. However, survival animals in control group did not exhibit renal clearance, contrasting with Lsa46 plus Lsa77 immunized group.

In fact, it seems that there is a synergistic effect when recombinant proteins are used in combination. The protective efficacy of rLp1454/rLp1118/rMceII combined proteins has been observed in several vaccine approaches [47–49]. The use of multicomponent vaccine is important not only to enhance efficacy, but also to promote cross protection against several serovars of* Leptospira*. Recently, a chimeric protein containing combined epitopes from OmpL1, LipL32, and LipL21 proteins was constructed and promising results were reported [50]. Accordingly, stimulating data have also been observed with another chimeric protein containing sequences of the LigA, Mce, Lsa45, OmpL1, and LipL41 proteins. The immunization with this chimeric protein plus MPLA adjuvant promoted higher titers of total IgG and IgG2/3 isotypes. In addition, sterilizing immunity was obtained in most immunized hamsters [25].

The immune response observed in the Lsa46+Lsa77-immunized group was similar to that found in the chimeric protein, showing a tendency towards a Th1 pattern. Even though the IgG subtypes in hamster are still not clear, some studies have showed that increased IgG2 antibodies is associated with the development of a Th1 response [51–53]. It was reported that type 1 response was necessary to prevent leptospiral renal colonization in cattle vaccinated with* L. borgpetersenii *serovar Hardjo infection [54, 55]. In this work, although the splenic lymphocyte proliferation and cytokine production have not been examined, in mouse model both proteins were able to induce Th1 cytokines, suggesting an ability to activate a cellular immune response [24].

Cell-mediated immunity is known to play an important role in control or clearance of infections with viruses and intracellular bacteria. However, IFN-*γ* produced by Th1 cells may also have a role in protection against extracellular microbes through its ability to activate macrophages and promote production of IgG2 classes of antibodies.* In vivo*, IFN-*γ* was required for nitric oxide production by macrophages against* Candida albicans, *an extracellular opportunistic pathogen [56]. Perhaps, antigens that are able to activate this branch of immune system are interesting targets for development of an effective vaccine against leptospirosis.

In conclusion, we characterized the capacity of Lsa46 and Lsa77 proteins to induce immune protection in hamsters against lethal infection. Although, these proteins individually were able to confer partial protection, survived hamsters presented renal carriage. A better performance was observed when both proteins were administered, with an increase rate of survival and a decrease of bacterial burden, though the data were not statistically significant. The use of new adjuvants or delivery vehicles should improve the immune protective potential of these proteins. Importantly, the present work demonstrates that the use of combined proteins may lead to the development of a vaccine capable of generating wide protection against pathogenic* Leptospira*.

## Figures and Tables

**Table 1:** Protein conservation among strains of *Leptospira*.

Gene	Protein given	Strain	Conservation %^2^
Locus*¹*	**name**		
LIC13479	**Lsa46**	*L. interrogans *serovar Copenhageni	100
		*L. interrogans *serovar Canicola	99
		*L. interrogans *serovar Icterohaemorrhagiae	100
		*L. interrogans *serovar Pomona	99
		*L. interrogans *serovar Hardjo	100
		*L. kirschneri * serovar Cynopteri	92
		*L. kirschneri * serovar Grippotyphosa	92
		*L. santarosai* serovar Shermani	77
		*L. noguchii* serovar Panama	88
		*L. borgpetersenii* serovar Hardjo-bovis	77
		*L.biflexa *serovar Patoc	45

LIC10050	**Lsa77**	*L. interrogans *serovar Copenhageni	100
		*L. interrogans *serovar Canicola	99
		*L. interrogans *serovar Icterohaemorrhagiae	100
		*L. interrogans *serovar Pomona	99
		*L. interrogans *serovar Hardjo	99
		*L. kirschneri * serovar Cynopteri	96
		*L. kirschneri * serovar Grippotyphosa	95
		*L. santarosai* serovar Shermani	89
		*L. noguchii* serovar Panama	96
		*L. borgpetersenii* serovar Hardjo-bovis	89
		*L.biflexa *serovar Patoc	44

*¹*http://aeg.ibi.ic.unicamp.br/world/lic/; LIC: *Leptospira interrogans* Copenhageni.

^2^http://blast.ncbi.nlm.nih.gov/Blast.cgi/.

**Table 2:** Effect of immunization with Lsa46 recombinant protein in a hamster model.

**Groups**	**N**°** Survivors / **		**N**°**Survivors/Total **	%** Total **	**Renal **	**Renal **	**Renal **	%
	**Total**			**Survival**	**Culture**	**Culture**	**Culture**	**Culture**
								**Positive**
					**Positive /**	**Positive /**	**Positive /**	
					**Total**	**Total**	**Total**	

	**Exp.1**	**Exp. 2**			**Exp. 1**	**Exp. 2**		

**PBS**	0/6	2/10	2/16	13	-	2/2	2/2	100

**Bacterin**	6/6	10/10	16/16	100	0/6	0/10	0/16	0

**Lsa46 +**	4/6	3/10	7/16	44	3/4	3/3	6/7	85
**Alum**								

**p-value** **∗**	0,03*∗*	0,5	0,056					

The statistical analysis refers to Fisher's exact test.*∗*p-values <0,05 were considered statistically significant.

**Table 3:** Effect of immunization with Lsa77 recombinant protein in a hamster model.

**Groups**	**N**°** Survivors/ **		**N**°**Survivors/Total**	%** Total **	**Renal **	**Renal **	**Renal **	%
	**Total**			**Survival**	**Culture**	**Culture**	**Culture**	**Culture**
								**Positive**
					**Positive / **	**Positive / **	**Positive / **	
					**Total**	**Total**	**Total**	

	**Exp.1**	**Exp. 2**			**Exp. 1**	**Exp. 2**		

**PBS**	2/10	5/8	7/18	39	2/2	0/5	2/7	28

**Bacterin**	10/10	6/6	16/16	100	0/10	0/6	0/16	0

**Lsa77 + **	5/10	4/8	9/18	50	4/5	0/4	4/9	44
**Alum**								

**p-value** **∗**	0,17	0,5	0,36					

The statistical analysis refers to Fisher's exact test.*∗*p-values <0,05 were considered statistically significant.

**Table 4:** Effect of immunization with Lsa46+Lsa77 recombinant proteins in a hamster model.

Groups	**N**°** Survivors/ **		**N**°**Survivors/**	%** Total **	**Renal **	**Renal **	**Renal **	%
	**Total**		**Total**		**Culture**	**Culture**	**Culture**	**Culture**
								**Positive**
				**Survival**	**Positive / **	**Positive / **	**Positive / **	
					**Total**	**Total**	**Total**	

	**Exp. 1**	**Exp. 2**			**Exp. 1**	**Exp. 2**		

**PBS**	4/6	3/6	7/12	58	2/4	3/3	5/7	71

**Bacterin**	6/6	6/6	12/12	100	0/6	0/6	0/12	0

**Lsa46 +**	6/6	5/6	11/12	90	2/6	3/5	5/11	45
**Lsa77 /**								
**Alum**								

**p-value** **∗**	0,22	0,27	0,07					

The statistical analysis refers to Fisher's exact test.*∗*p-values <0,05 were considered statistically significant.

## Data Availability

The data used to support the findings of this study are available from the corresponding author upon request.

## References

[1] Costa F., Hagan J. E., Calcagno J. (2015). Global morbidity and mortality of leptospirosis: a systematic review. *PLOS Neglected Tropical Diseases*.

[2] Costa F., Ribeiro G. S., Felzemburgh R. D. M. (2014). Influence of Household Rat Infestation on Leptospira Transmission in the Urban Slum Environment. *PLOS Neglected Tropical Diseases*.

[3] Bharti A. R., Nally J. E., Ricaldi J. N. (2003). Leptospirosis: a zoonotic disease of global importance. *The Lancet Infectious Diseases*.

[4] Faine S., Adler B., Bolin C., Perolat P. (1999). *Leptospira and Leptospirosis*.

[5] Yan Y., Chen Y., Liou W. (2003). An evaluation of the serological and epidemiological effects of the outer envelope vaccine to leptospira. *Journal of the Chinese Medical Association*.

[6] Martínez R., Pérez A., Quiñones M. d. (2004). Efficacy and safety of a vaccine against human leptospirosis in Cuba. *Revista Panamericana de Salud Pública/Pan American Journal of Public Health*.

[7] Laurichesse H., Gourdon F., Smits H. L. (2007). Safety and immunogenicity of subcutaneous or intramuscular administration of a monovalent inactivated vaccine against *Leptospira interrogans* serogroup Icterohaemorrhagiae in healthy volunteers. *Clinical Microbiology and Infection*.

[8] Adler B., de la Peña Moctezuma A. (2010). Leptospira and leptospirosis. *Veterinary Microbiology*.

[9] Odir A. D., Grassmann A. A., Hartwig D. D., Félix S. R., Da Silva É. F., McBride A. J. A. (2011). Recombinant vaccines against leptospirosis. *Human Vaccines & Immunotherapeutics*.

[10] Grassmann A. A., Félix S. R., Dos Santos C. X. (2012). Protection against lethal leptospirosis after vaccination with LipL32 coupled or coadministered with the b subunit of escherichia coli heat-labile enterotoxin. *Clinical and Vaccine Immunology*.

[11] Atzingen M. V., Vieira M. L., Oliveira R. (2012). Evaluation of immunoprotective activity of six leptospiral proteins in the hamster model of leptospirosis. *The Open Microbiology Journal*.

[12] Humphryes P. C., Weeks M. E., AbuOun M., Thomson G., Núñez A., Coldham N. G. (2014). Vaccination with leptospiral outer membrane lipoprotein LipL32 reduces kidney invasion of Leptospira interrogans serovar canicola in hamsters. *Clinical and Vaccine Immunology*.

[13] Oliveira T. L., Grassmann A. A., Schuch R. A. (2015). Evaluation of the leptospira interrogans outer membrane protein OmpL37 as a vaccine candidate. *PLoS ONE*.

[14] Monaris D., Sbrogio-Almeida M. E., Dib C. C. (2015). Protective immunity and reduced renal colonization induced by vaccines containing recombinant Leptospira interrogans outer membrane proteins and flagellin adjuvant. *Clinical and Vaccine Immunology*.

[15] Evangelista K. V., Lourdault K., Matsunaga J., Haake D. A. (2017). Immunoprotective properties of recombinant LigA and LigB in a hamster model of acute leptospirosis. *PLoS ONE*.

[16] Conrad N. L., Cruz McBride F. W., Souza J. D. (2017). LigB subunit vaccine confers sterile immunity against challenge in the hamster model of leptospirosis. *PLOS Neglected Tropical Diseases*.

[17] Koizumi N., Watanabe H. (2004). Leptospiral immunoglobulin-like proteins elicit protective immunity. *Vaccine*.

[18] Palaniappan R. U. M., McDonough S. P., Divers T. J. (2006). Immunoprotection of recombinant leptospiral immunoglobulin-like protein A against Leptospira interrogans serovar pomona infection. *Infection and Immunity*.

[19] Silva É. F., Medeiros M. A., McBride A. J. A. (2007). The terminal portion of leptospiral immunoglobulin-like protein LigA confers protective immunity against lethal infection in the hamster model of leptospirosis. *Vaccine*.

[20] Yan W., Faisal S. M., McDonough S. P. (2009). Immunogenicity and protective efficacy of recombinant Leptospira immunoglobulin-like protein B (rLigB) in a hamster challenge model. *Microbes and Infection*.

[21] Lourdault K., Wang L.-C., Vieira A. (2014). Oral immunization with escherichia coli expressing a lipidated form of LigA protects hamsters against challenge with leptospira interrogans serovar Copenhageni. *Infection and Immunity*.

[22] Bacelo K. L., Hartwig D. D., Seixas F. K. (2014). Xanthan gum as an adjuvant in a subunit vaccine preparation against leptospirosis. *BioMed Research International*.

[23] Dellagostin O. A., Grassmann A. A., Rizzi C. (2017). Reverse vaccinology: An approach for identifying leptospiral vaccine candidates. *International Journal of Molecular Sciences*.

[24] Teixeira A. F., De Morais Z. M., Kirchgatter K., Romero E. C., Vasconcellos S. A., Nascimento A. L. T. O. (2015). Features of two new proteins with OmpALike domains identified in the genome sequences of Leptospira interrogans. *PLoS ONE*.

[25] Fernandes L. G. V., Teixeira A. F., Filho A. F. S. (2017). Immune response and protective profile elicited by a multi-epitope chimeric protein derived from Leptospira interrogans. *International Journal of Infectious Diseases*.

[26] Gómez R. M., Vieira M. L., Schattner M. (2008). Putative outer membrane proteins of Leptospira interrogans stimulate human umbilical vein endothelial cells (HUVECS) and express during infection. *Microbial Pathogenesis*.

[27] Evangelista K., Franco R., Schwab A., Coburn J. (2014). Leptospira interrogans binds to cadherins.. *PLOS Neglected Tropical Diseases*.

[28] Siqueira G. H., Teixeira A. F., Fernandes L. G. (2016). The recombinant LIC10508 is a plasma fibronectin, plasminogen, fibrinogen and C4BP-binding protein of Leptospira interrogans. *Pathogens and Disease*.

[29] Matsunaga J., Barocchi M. A., Croda J. (2003). Pathogenic Leptospira species express surface-exposed proteins belonging to the bacterial immunoglobulin superfamily. *Molecular Microbiology*.

[30] Faisal S. M., Yan W., Chen C.-S., Palaniappan R. U. M., McDonough S. P., Chang Y.-F. (2008). Evaluation of protective immunity of Leptospira immunoglobulin like protein A (LigA) DNA vaccine against challenge in hamsters. *Vaccine*.

[31] Coutinho M. L., Choy H. A., Kelley M. M. (2011). A ligA three-domain region protects hamsters from lethal infection by leptospira interrogans. *PLOS Neglected Tropical Diseases*.

[32] Deveson Lucas D. S., Cullen P. A., Lo M., Srikram A., Sermswan R. W., Adler B. (2011). Recombinant LipL32 and LigA from Leptospira are unable to stimulate protective immunity against leptospirosis in the hamster model. *Vaccine*.

[33] McBride A. J. A., Cerqueira G. M., Suchard M. A. (2009). Genetic diversity of the Leptospiral immunoglobulin-like (Lig) genes in pathogenic Leptospira spp.. *Infection, Genetics and Evolution*.

[34] Cao Y., Faisal S. M., Yan W. (2011). Evaluation of novel fusion proteins derived from extracellular matrix binding domains of LigB as vaccine candidates against leptospirosis in a hamster model. *Vaccine*.

[35] Forster K. M., Hartwig D. D., Seixas F. K. (2013). A conserved region of leptospiral immunoglobulin-like A and B proteins as a DNA vaccine elicits a prophylactic immune response against leptospirosis. *Clinical and Vaccine Immunology*.

[36] Atzingen M. V., Gonçales A. P., De Morais Z. M. (2010). Characterization of leptospiral proteins that afford partial protection in hamsters against lethal challenge with Leptospira interrogans. *Journal of Medical Microbiology*.

[37] Weiser J. N., Gotschlich E. C. (1991). Outer membrane protein A (OmpA) contributes to serum resistance and pathogenicity of Escherichia coli K-1. *Infection and Immunity*.

[38] Prasadarao N. V., Wass C. A., Weiser J. N., Stins M. F., Huang S.-H. E., Kim K. S. (1996). Outer membrane protein A of Escherichia coli contributes to invasion of brain microvascular endothelial cells. *Infection and Immunity*.

[39] Prasadarao N. V., Blom A. M., Villoutreix B. O., Linsangan L. C. (2002). A novel interaction of outer membrane protein A with C4b binding protein mediates serum resistance of Escherichia coli K1. *The Journal of Immunology*.

[40] Guan Q., Wang X., Wang X. (2015). Recombinant outer membrane protein A induces a protective immune response against Escherichia coli infection in mice. *Applied Microbiology and Biotechnology*.

[41] Simborio H. L. T., Reyes A. W. B., Hop H. T. (2015). Immune modulation of recombinant OmpA against Brucella Abortus 544 infection in mice. *Journal of Microbiology and Biotechnology*.

[42] Zhang X., Yang T., Cao J., Sun J., Dai W., Zhang L. (2016). Mucosal immunization with purified OmpA elicited protective immunity against infections caused by multidrug-resistant Acinetobacter baumannii. *Microbial Pathogenesis*.

[43] Yan W., Faisal S. M., McDonough S. P. (2010). Identification and characterization of OmpA-like proteins as novel vaccine candidates for Leptospirosis. *Vaccine*.

[44] Seixas F. K., Fernandes C. H., Hartwig D. D., Conceição F. R., Aleixo J. A. G., Dellagostin O. A. (2007). Evaluation of different ways of presenting LipL32 to the immune system with the aim of developing a recombinant vaccine against leptospirosis. *Canadian Journal of Microbiology*.

[45] Branger C., Sonrier C., Chatrenet B. (2001). Identification of the hemolysis-associated protein 1 as a cross-protective immunogen of Leptospira interrogans by adenovirus-mediated vaccination. *Infection and Immunity*.

[46] Branger C., Chatrenet B., Gauvrit A. (2005). Protection against Leptospira interrogans sensu lato challenge by DNA immunization with the gene encoding hemolysin-associated protein 1. *Infection and Immunity*.

[47] Chang Y.-F., Chen C.-S., Palaniappan R. U. M. (2007). Immunogenicity of the recombinant leptospiral putative outer membrane proteins as vaccine candidates. *Vaccine*.

[48] Faisal S. M., Yan W., McDonough S. P., Mohammed H. O., Divers T. J., Chang Y.-F. (2009). Immune response and prophylactic efficacy of smegmosomes in a hamster model of leptospirosis. *Vaccine*.

[49] Faisal S. M., Yan W., McDonough S. P., Chang C.-F., Pan M.-J., Chang Y.-F. (2009). Leptosome-entrapped leptospiral antigens conferred significant higher levels of protection than those entrapped with PC-liposomes in a hamster model. *Vaccine*.

[50] Lin X., Xiao G., Luo D. (2016). Chimeric epitope vaccine against Leptospira interrogans infection and induced specific immunity in Guinea pigs. *BMC Microbiology*.

[51] Bhowmick S., Ravindran R., Ali N. (2007). Leishmanial antigens in liposomes promote protective immunity and provide immunotherapy against visceral leishmaniasis via polarized Th1 response. *Vaccine*.

[52] Shivahare R., Vishwakarma P., Parmar N. (2014). Combination of liposomal CpG oligodeoxynucleotide 2006 and miltefosine induces strong cell-mediated immunity during experimental visceral leishmaniasis. *PLoS ONE*.

[53] Verma R., Joseph S. K., Kushwaha V. (2015). Cross reactive molecules of human lymphatic filaria Brugia malayi inhibit Leishmania donovani infection in hamsters. *Acta Tropica*.

[54] Naiman B. M., Alt D., Bolin C. A., Zuerner R., Baldwin C. L. (2001). Protective killed Leptospira borgpetersenii vaccine induces potent Th1 immunity comprising responses by CD4 and *γδ* T lymphocytes. *Infection and Immunity*.

[55] Brown R. A., Blumerman S., Gay C., Bolin C., Duby R., Baldwin C. L. (2003). Comparison of three different leptospiral vaccines for induction of a type 1 immune response to Leptospira borgpetersenii serovar Hardjo. *Vaccine*.

[56] Káposzta R., Tree P., Maródi L., Gordon S. (1998). Characteristics of invasive candidiasis in gamma interferon- and interleukin-4-deficient mice: Role of macrophages in host defense against candida albicans. *Infection and Immunity*.

